# Diagnostic challenges for a case of maxillary carcinoma cuniculatum in the setting of human immunodeficiency virus: a case report

**DOI:** 10.1007/s10006-026-01562-4

**Published:** 2026-04-24

**Authors:** Jeremy X. Figueroa-Ortiz, Kale McMillan, Jonathan B. McHugh, Derek F. Holecek

**Affiliations:** 1 Department of Oral and Maxillofacial Surgery, University of Michigan, 1515 E Hospital Dr, SPC 5222, Towsley Center Ste G1100, Ann Arbor, MI 48109 USA; 2 Department of Pathology, University of Michigan, Ann Arbor, MI USA

**Keywords:** Carcinoma cuniculatum, Squamous cell carcinoma, Immune dysregulation, Osteomyelitis, Verrucous Carcinoma

## Abstract

**Purpose:**

Carcinoma Cuniculatum (CC) is an exceptionally rare variant of squamous cell carcinoma with limited description in the literature. Oral Carcinoma Cuniculatum (OCC) continues to be a diagnostic dilemma as it generally presents as an indolent infection, often undergoing multiple biopsies and debridements prior to definitive diagnosis. This study aims to review the diagnostic challenges, clinical features, histopathology, and management of OCC. We specifically present a unique case of OCC in a patient with human immunodeficiency virus (HIV).

**Methods:**

A review of the current literature on OCC was conducted, focusing on diagnostic delays, clinical presentation, histopathologic characteristics, and treatment approaches. Additionally, a case report of a 47-year-old male with OCC is presented, including clinical course, diagnostic workup, and management.

**Results:**

The patient underwent multiple biopsies and surgical debridements over a 3-year period prior to definitive diagnosis. He was ultimately treated with subtotal maxillectomy and radial forearm free flap reconstruction. Final pathology demonstrated pT4a OCC. His clinical course was further complicated by previously undiagnosed HIV infection. Multidisciplinary tumor board recommendations included bilateral elective neck dissections and adjuvant radiation therapy.

**Conclusion:**

OCC remains a diagnostic challenge due to its indolent behavior and nonspecific presentation, often resulting in delayed diagnosis and advanced disease at treatment. This case highlights the importance of maintaining clinical suspicion for malignancy in persistent oral lesions and may represent the first reported case of OCC in a patient with HIV, thus raising new questions regarding the potential role of immunosuppression while propagating prior inquiries.

## Introduction

Carcinoma Cuniculatum (CC) is a rare locally destructive subtype of squamous cell carcinoma (SCC) with limited description throughout the literature. It was first described in the mid 20th century, where physicians reported rare cases of invasive tumors of the foot with malodorous exudate and no clear histopathologic diagnosis in the setting of multiple biopsies. After a robust case series clinicians were able to compile sufficient histopathologic evidence to establish this rare subtype of squamous malignancy [1]. CC has since been discovered in other anatomic areas, including the oral cavity [2].

Oral carcinoma cuniculatum (OCC) remains a diagnostic challenge. Its non-specific presentation often mirrors indolent infection requiring multiple debridements and biopsies prior to definitive diagnosis. As noted in a systematic review by Farag et al., all described cases of OCC were misdiagnosed at initial biopsy [3]. It is estimated that its current prevalence amongst SCC is 1.9–2.7% [4, 5], which is opined to be deceptively low due to this diagnostic difficulty. Our aim is to describe a novel case of maxillary OCC in the setting of severe immunosuppression, common clinical and histopathologic challenges, and provide a brief review of literature.

### Case Report

A 47-year-old male with newly diagnosed human immunodeficiency virus (HIV), type II diabetes mellitus (A1c 6.9%), and remote 22.5 pack year tobacco history, was referred to the clinic at the University of Michigan by for consultation of a maxillary lesion.

The patient describes a three-year course of cyclical symptoms prompted by placement and subsequent failure of dental implant at site #10. Due to progressive osteolysis, the patient underwent several biopsies, multiple extensive debridements, sinus exploration, and extraction of multiple teeth guided by repeat imaging. Multiple bone biopsies were obtained concerning for osteomyelitis per outside pathology reports. Culture data guided several courses of antibiotics by infectious disease (ID) physicians. On final debridement by referring Oral & Maxillofacial Surgeon (OMS), biopsy yielded similar bony changes with new, non-specific soft tissue finding of verrucous squamoproliferative component, prompting referral to our center.

**Fig. 1:**
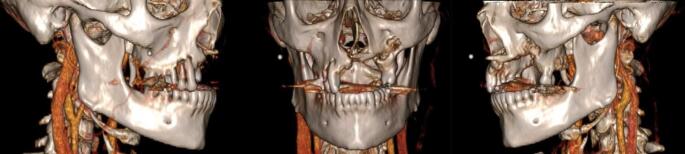
Preoperative three-dimensional reconstruction visualizing maxillary defect in sequential views—following debridements before composite resection

Clinical examination confirmed ongoing purulent exudate with signs of inflammation, including erythematous mucosa with areas of exposed necrotic bone. Repeat computed tomograms (CT) with intravenous (IV) contrast revealed erosive alveolar defects with extension to maxillary sinus (Fig. 1) with non-aggressive maxillary mucosal thickening.

**Fig. 2:**
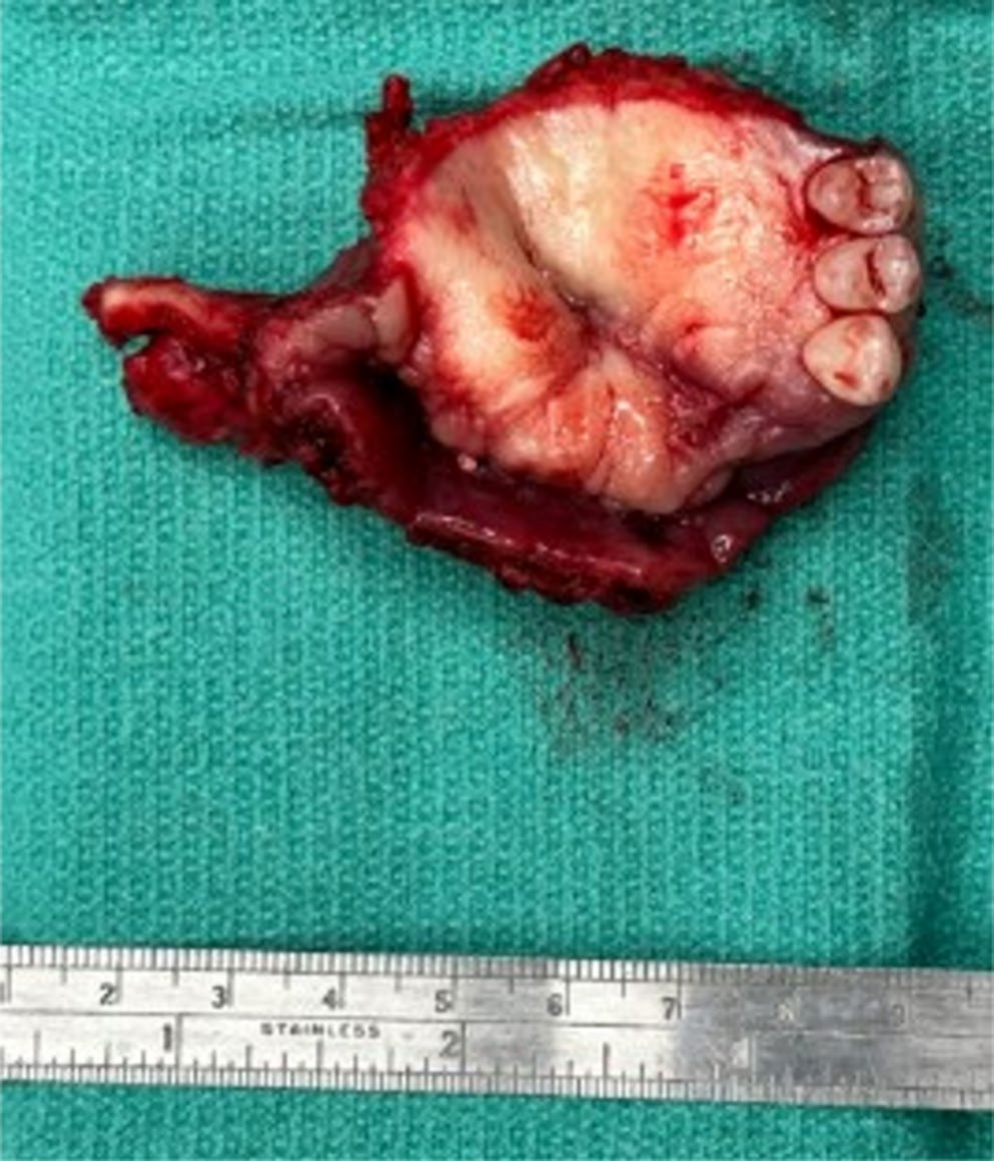
Subtotal maxillectomy specimen of anterior maxilla, with evidence of limited erythematous cobble stoning and burrowing ulceration along the alveolar ridge

The clinical presentation, radiographic imaging, and repeated histopathologic analyses were consistent with progressive infection and bone necrosis, therefore osteomyelitis was included on the differential diagnosis. We proceeded with a subtotal maxillectomy and coverage with radial forearm free flap, followed by prolonged course of postoperative IV antibiotics guided by the ID team. Given the clinically noted, boggy erythematous overlying gingiva, and prior biopsy with verrucous changes, composite resection of overlying gingival tissue was included for additional histopathologic analysis (Fig. 2). All intraoperative frozen section margins were negative for malignancy.

On permanent histopathologic analysis, the specimen exhibited features of OCC with subsequent 1.2 cm depth of invasion, and bone invasion, subsequently staged as a pT4a maxillary malignancy. After presentation at multidisciplinary tumor board, it was recommended the patient undergo bilateral elective neck dissections (END) and adjuvant radiation therapy (RT). The patient elected to defer surgical neck management but proceeded with RT. He initiated adjuvant RT at 6 weeks post operatively, and received a total of 60gy, delivered over 30 fractions. He has followed surveillance recommendations per oral squamous cell carcinoma (OSCC) guidelines by the National Comprehensive Cancer Network (NCCN) with 3 month surveillance follow up. His post treatment CT imaging up to 1 year post operatively shows no evidence of new or recurrent disease. See Table 1 for brief summary of clinical course.

**Table 1:** Summary of clinical course

T_0_	Clinical Course	
	• Initial presentation for failed dental implant at site #10 • Debridement, biopsy	
T_1_	• T_0_ + 36 months • Newly diagnosed HIV • Concern for malignancy, biopsy resulted non-vital bone, chronic inflammation and reactive mucosa • Candidiasis noted	
T_2_	• T_1_ + 2 months • Extraction of teeth #9, 11–14 with extensive maxillary sinus debridement	
T_3_	• T_2_ + 3 months • Extraction of teeth #7, 8, debridement of maxilla and palate • Bone with reported osteomyelitic appearance	
T_4_	• T_3_ + 1 month • Debridement and biopsy revealing squamous mucosa with verrucoid squamoproliferative lesion • Moderate growth of yeast • Referral placed for further management	
T_5_	• T_4_ + 2 months • Operative subtotal maxillectomy • Histopathology consistent with OCC • Negative margins, no perineural invasion	

### Clinical Presentation of Carcinoma Cuniculatum

The clinical presentation and etiology of OCC is poorly characterized in the literature, limited by low disease prevalence [3, 6]. Clinical variance of OCC has contributed to its misdiagnosis as osteomyelitis, benign cystic lesions, verrucous carcinoma (VC), or oral squamous cell carcinoma (OSCC). Farag et al. highlights the disease variations in the largest review of OCC reported in the English literature with a subtotal of 43 cases (Table 2).

**Table 2:** Summary of OCC literature review and applicable treatment methods

Author (Year)	Study Type / Sample	Key Findings (Diagnosis Challenges)	Treatment Approach / Outcomes
Aird et al. [1]	Original description (cutaneous)	First identification; deeply burrowing growth pattern linked to diagnostic confusion	Surgical excision established as primary treatment
Flieger & Owiński [2]	Early oral case report	Rare oral presentation; frequently misidentified due to unfamiliarity	Surgical management
Farag et al. [3]	Systematic review	Often misdiagnosed as verrucous carcinoma or benign lesions; biopsy may be non-representative	Wide surgical excision preferred; low metastasis risk
Barrett et al. [4]	Case series (12 cases)	Histopathological overlap with cystic SCC; sampling errors common	Emphasizes need for complete excision and accurate pathology
Sun et al. [5]	Clinicopathologic study	Bland cytology despite invasive behavior → underdiagnosis	Surgery effective; good prognosis if fully removed
Yadav et al. [6]	Case series (6 cases)	Deep sinus tracts and keratin-filled channels often missed in superficial biopsies	Surgical resection; favorable outcomes
Hutton et al. [7]	Pediatric case	Atypical age complicates diagnosis; rarity leads to delayed recognition	Surgical excision successful
Padilla & Murrach [9]	Case report/review	Underdiagnosed without clinicopathologic correlation	Highlights importance of correlating imaging and histology
Shapiro et al. [10]	Case report	Can cause significant bone destruction despite benign histologic appearance	Aggressive surgical resection required
Elangovan et al. [11]	Case report	Mimics other oral lesions (e.g., verrucous carcinoma, leukoplakia)	Surgical management; emphasizes early diagnosis
Allon et al. [12]	Case series	Diagnostic delay due to rarity and indolent course	Surgical excision
Pons et al. [13]	Case series (3 cases)	Radiographic findings resemble osteomyelitis or complex cystic lesions	Mandibular resection when bone involved
Nagai et al. [14]	Case report with literature review	Diagnostic challenge due to deceptively benign histology	Surgery mainstay; avoid overtreatment with radiotherapy
Vered & Wright [15]	WHO update	Classification clarifies entity but still underrecognized	Reinforces surgery as standard
Abilasha et al. [16]	Case report	Some cases show aggressive behavior → diagnostic underestimation	Aggressive surgical approach needed
Johnson et al. [17]	Classification reference	Helps differentiate from other SCC variants	Focuses on standard SCC-based treatment principles
Kruse & Graetz [24]	Case report	Diagnostic confusion with verrucous carcinoma	Surgical excision
Massé et al. [25]	Recent review	Continues to be underrecognized; highlights need for deep biopsy	Surgery remains gold standard
Suzuki et al. [27]	Case report	Mimics leukoplakia clinically	Surgical excision

The predominant age of presentation is within the sixth and seventh decades of life. There is a single case report of a 7-year-old girl [7], however, this remains anomalous. Unlike OSCC, OCC has a slightly stronger predilection for females. Only one third of OCC cases report tobacco or heavy alcohol use [3, 8]. Presenting symptoms are vague: including pain, soft tissue ulceration, swelling, and induration. On clinical examination, OCC tends to be sessile, with flesh color or erythematous hues. There may be mild papillary projections or cobblestoning. Although OSCC can have a similar presentation, VC will typically present as a leukoplakic lesion with prominent surface projections [9, 10]. Interestingly, approximately 16% of OCC are reported to have exudate, parallelling suppurative chronic osteomyelitis. The ongoing symptoms coupled with the prolonged duration of lesions, which is on average 19 months in duration prior to diagnosis and definitive management [3], mirrors a chronic infectious etiology or a more indolent malignancy, such as VC. Regarding anatomic distribution, approximately half of reported cases in the mandibular gingiva, approximately 25% in the tongue, and only 13% in maxillary gingiva.

Previous authors note varying patterns of bony penetration on radiographic imaging, which could provide diagnostic clues: VC pushes against the bony surface with associated resorption, whereas OCC and SCC are more prone to cause large, erosive cavities [11]. This method is not data driven and is unreliable for diagnosis. With consistency, much of the literature describes ill-defined osteolytic changes with cortical destruction, which is non-specific as it can be found in inflammatory conditions as well as malignant pathology, in which most authors recommend clinical and histopathologic correlation [7, 12, 13].

### Histopathology

OCC’s presenting profile emphasizes the need for adequate biopsy specimen for accurate histopathologic review. Inadequate depth on biopsy can lead to an inadequate specimen [14], however, even an extensive specimen does not guarantee ease of diagnosis, as exhibited in this case review.

Gross specimen of OCC is generally described as granular or papillomatous. Histologically, OCC appears well-differentiated, lacking cellular atypia, with a penetrating pattern of growth. This produces the hallmark deep branching and keratin-filled crypts resembling ‘rabbit burrows’. Most specimens develop cryptic micro-abscesses, producing a purulent exudate inoculated with neutrophils [15]. Bony sequestrae are thought to reflect bony invasion. There have been reports of more aggressive variants of CC throughout the body, with features of cellular cannibalism, however, there is only one such reported case in OCC [16].

In contrast, VC and OSCC lack the complex channeling and branching of OCC. OSCC tends to be aggressive and variably differentiated. VC can possess epithelial clefting and parakeratin plugging, but is more superficial with broad advancement, which lends as a factor of differentiation [11].

**Fig. 3:**
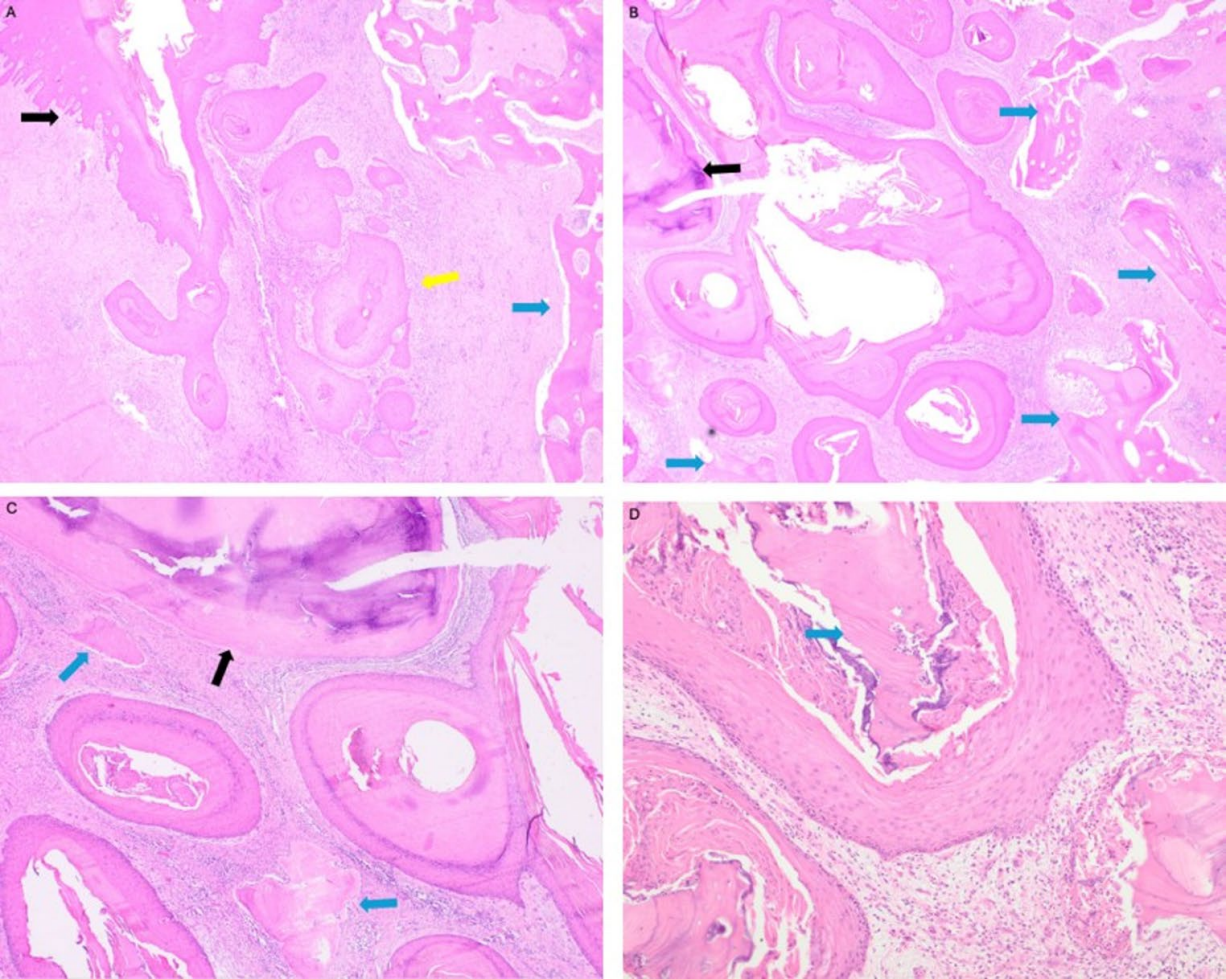
Operative specimen: Subtotal Maxillectomy with associated soft tissue cuff. Hematoxylin and Eosin stain. **(A)** Burrow-like endophytic invaginations (yellow) of well-differentiated SCC extending from surface (black) and into bone (blue). **(B)** Infiltrative cystic keratin-filled crypts infiltrating maxillary bone (blue) and adjacent tooth (black). **(C)** Infiltrative cystic keratin-filled crypts infiltrating maxillary bone (blue) and adjacent tooth (black). **(D)** Island of well-differentiated SCC with central cysts, neutrophils, and necrotic bone (blue)

In the case presented, several biopsies demonstrated non-malignant discovery. A 3.8 cm specimen was analyzed and discovered to have classic characteristics of OCC (Fig. 3). The case report being described, as well as others in the literature, emphasizes the diagnostic complexity, aligning the WHO disposition on correlation with clinical and radiographic features being required [17].

### Immune dysregulation

Immune dysregulation is well described in oncologic pathogenesis [18, 19]. This is seen specifically in oropharyngeal squamous cell carcinoma (OPSCC), Kaposi’s Sarcoma, and non-Hodgkin’s lymphomas [20]. In contrast, there has been no strong correlations to immunosuppression or well-defined tumor markers in OCC. This gains relevance as this is the first documented case of OCC in an HIV+ patient, who was undiagnosed for the vast duration of their clinical course.

Previous case reports discussing esophageal CC have mentioned a possible correlation with immunosuppression [21], however, evidence is limited. Authors have evaluated cell cycle disruption and known markers of malignancy (p53, Ki-67) without establishing any direct correlation in OCC. Although some studies show correlation of human papillomavirus (HPV) with cutaneous CC [22], however, this has not translated to OCC.

### Prognostication and management

Treatment of OCC depends on staging, which follows standard American Joint Committee on Cancer (AJCC) guidelines. Primary management is surgical resection with negative margins. There is emphasis on adequate resection due to concern for anaplastic transformation with repeated surgical intervention [23]. While 1 cm margins are typically considered appropriate, there is no consensus on recommended surgical or histopathologic margins.

There continues to be significant debate regarding the therapeutic benefit of END. This discussion derives from the nature of OCC as it is locally invasive with minimal and rare propensity to metastasize [3, 5, 10, 24]. Although many case reports have been managed with END, there is no data supporting a survival benefit [24, 25]. Similar discourse exists regarding the benefits of adjuvant radiation therapy (RT). As OCC exhibits locally aggressive behavior, some believe RT is warranted, while others argue that adjuvant RT may provoke anaplastic transformation [26]. Overall, surgical management of the neck and adjuvant RT is determined on a case-by-case basis with a tendency to follow national guidelines for locoregional control and multidisciplinary tumor board review.

Prognostication is favorable with excellent survivorship and rare metastasis or recurrence, with an estimated 2- and 5-year survival of 93.3% and 85.7%, respectively^5^. Mortality is attributed to tumor size and destruction of local structures, rather than metastasis [3, 23, 27]. This only emphasizes the need for adequate resection. Specific guidelines would aid standardized management, but low disease incidence with poor understanding of the pathogenesis makes this difficult.

## Conclusion

As shown in this case report and noted in multiple previous studies, OCC continues to be a diagnostic challenge. The indolent nature of the disease, often presenting in a similar manner to osteomyelitis, prompting multiple surgical interventions, often complicates the diagnostic picture. In summary, this is a case report of a rare malignancy, in a rare anatomic site, with symptoms precipitated via dental implant placement, in the setting of long-standing undiagnosed immunosuppression. There has yet to be a similar case reported in the literature. This case poses new questions regarding the role of immunosuppression while propagating prior inquiries—true incidence, prevalence, pathogenesis, role of surgery in progression and transformation, as well as the development of evidence-based management.

## Data Availability

No datasets were generated or analysed during the current study.
